# ECMO in paediatric septic shock: an urgent need for prospective trial

**DOI:** 10.1186/s13054-020-2789-7

**Published:** 2020-03-12

**Authors:** Xavier Beretta-Piccoli, Dominique Biarent, David De Bels, Patrick M. Honore, Sébastien Redant

**Affiliations:** 1 ICU Department, Hopital Universitaire des Enfants Reine Fabiola HUDERF, Université libre de Bruxelles, ULB, Brussels, Belgium; 2 ICU Department, Centre Hospitalier Universitaire Brugmann, Brugmann University Hospital, Place Van Gehuchtenplein,4, 1020 Brussels, Belgium

The publication by Schlapbach et al. used a sepsis mortality prediction model to define a mortality threshold above which patients suffering from septic shock could benefit from venoarterial ECMO (VA-ECMO). When predicted mortality was lower than 47.1%, the measured mortality was 11.8% higher, while when the predicted mortality was higher than 47.1%, the measured mortality was 16.2% lower. Seventy-one per cent of patients were cannulated centrally with a central flow significantly higher than peripheral ECMO (173 versus 129 ml/kg/min, *p* < 0.05) [1]. However, central techniques are not available everywhere, and percutaneous VA-ECMO technique is spreading and frequently done by intensivists but does not allow flow as high as those described by Schlapbach. We were surprised by the benefit brought by central cannulation on survival in the multivariate analysis (*p* = 0.046) while higher flows did not reach significance on survival nor in the uni- (*p* = 0.27) nor in the multivariate analyses (*p* = NS). Other studies in paediatric septic shock have shown a survival benefit from ECMO flow rates greater than 150 ml/kg/min [2].

The mortality score developed for the study is simple and practical. However, it does not distinguish between the different haemodynamic profiles seen in paediatric shock. More than 50% of children with septic shock have cold clamped extremities, low cardiac output and elevated vascular resistance [3]. VA-ECMO in this profile may restore cardiac output hence improving haemodynamics. The debate remains open in high cardiac output shock where the native heart delivers a flow greater than the maximal peripheral ECMO flow. The guidelines recommend VA-ECMO in refractory shock with a special attention to the cardiac index. When it is less than 3.3 l/min/m^2^, optimizing inotropes should be first considered before ECMO [4].

As proposed by the authors, prospective studies are necessary, and we suggest to include ultrasound values such as the cardiac index, the systemic vascular resistance index and the velocity time integral [5]. Even if it has been shown that these parameters are not interpretable on admission because they are preload-dependent, once the active filling phase has been completed, the echocardiography is a tool which enables the practitioner to be informed on the exact haemodynamic profile of the child [5]. The question that a prospective study should answer is whether a particular haemodynamic profile will better answer to VA-ECMO and accordingly which ECMO flow will be appropriate.

## Authors’ response

Luregn J Schlapbach^1,2,3^, Warwick Butt^4^, Graeme MacLaren^4,5^

on behalf of the Australian & New Zealand Intensive Care Society (ANZICS) Paediatric Study Group

^1^Paediatric Critical Care Research Group, Child Health Research Centre, The University of Queensland, Brisbane, Australia

^2^Paediatric Intensive Care Unit, Queensland Children’s Hospital, Brisbane, Australia

^3^Department of Intensive Care Medicine and Neonatology, and Children's Research Center, University Children's Hospital of Zurich, University of Zurich, Zurich, Switzerland.

^4^Paediatric Intensive Care Unit, The Royal Children’s Hospital, Melbourne, Australia

^5^Cardiothoracic Intensive Care Unit, National University Health System, Singapore, Singapore

We thank Beretta-Piccoli and colleagues for their comment regarding our study defining benefit thresholds for ECMO in children with septic shock [1]. The 2020 Pediatric Surviving Sepsis Campaign [6] guidelines issued a recommendation based on low-quality evidence for venoarterial ECMO as rescue therapy for septic shock “refractory to all other treatments”. The problem is when to consider shock “refractory” [7]. The mortality in refractory shock even without ECMO is not 100%, and clinicians have to weigh risk versus benefit of ECMO. While every day clinical decision-making embraces implicit or explicit benefit threshold estimates, our study provides one of the first objective approaches to this scenario.

The first point raised by Beretta-Piccoli et al. relates to the generalizability of the findings, given that central cannulation was the predominant access used in our study. Central cannulation provides higher flows and maintains bilateral carotid perfusion with potentially lower cerebral stroke incidence at the expense of higher overall bleeding and infection risk. While central ECMO currently is limited to cardiosurgical centres, these complications are manageable, and timely referral to such high-volume centres should be emphasized.

The second point makes reference to the fact that the Paediatric Sepsis Score [8] used in the study provides no direct measure of the haemodynamic pattern. Yet, cold shock or warm shock, respectively, are not fixed entities but rather reflect a dynamic process: Septic children often move from hyperdynamic states with variable systemic vascular resistance to progressive cardiogenic shock. Regardless of the pattern, ECMO with appropriate flows can reverse the deficit in oxygen delivery to tissues. Sequential rather than single assessment of the Sepsis Score may improve discrimination on children on a trajectory towards cardiac arrest and irreversible organ damage.

Finally, Beretta-Piccoli et al. propose an ultrasound-based study assessing haemodynamic profiles to assist in decisions on ECMO initiation and to tailor the cannulation strategy based on the required flows. We concur that repeated echocardiographic assessment of cardiac function should be routinely performed. Accurate and rapid assessment can improve the timeliness of cannulation. Although the highest ECMO benefit was observed in children post-cardiac arrest, clinicians should endeavour to recognize impending cardiac arrest prior to its occurrence [2].

In summary, although decisions on whether to place a child with sepsis on ECMO may always happen on an individual basis, such should not prevent us from applying benefit threshold estimates to assess the appropriateness of such decisions—similar to most areas of critical care where risk-adjusted benchmarking has been the standard for decades [9].

## Data Availability

Not applicable.

## Abbreviations

VA-ECMO: Venoarterial ECMO
